# Risk factors and clinical outcome of postoperative hyperglycemia after cardiac surgery with cardiopulmonary bypass

**DOI:** 10.3389/fcvm.2025.1479922

**Published:** 2025-04-07

**Authors:** Yuping Xiang, Tianhui Luo, Ling Zeng

**Affiliations:** Department of Critical Care Medicine, West China Hospital, Sichuan University/West China School of Nursing, Sichuan University, Chengdu, Sichuan, China

**Keywords:** cardiac surgery, cardiopulmonary bypass, postoperative hyperglycemia, risk factors, clinical outcomes

## Abstract

**Background:**

There is a high incidence of postoperative hyperglycemia (PHG) in cardiac surgery with cardiopulmonary bypass (CPB), as well as increased morbidity and mortality. The purpose of this study was to evaluate the incidence of PHG after cardiac surgery with CPB, the independent risk factors, and its association with clinical outcomes.

**Methods:**

This was a retrospective, observational study of patients who underwent cardiac surgery with CPB between January 2023 and March 2024 in West China Hospital of Sichuan University. A total of 1,008 consecutive postoperative cardiac surgery patients admitted to the cardiac surgery intensive care unit (ICU) were divided into a non-PHG group and a PHG group. Patients’ blood glucose levels were evaluated immediately after cardiac surgery and every 3–4 h daily for 10days, until discharge from the ICU. For patients with PHG, intravenous insulin infusion was performed according to the institution's protocol, and perioperative risk factors for hyperglycemia and clinical outcomes were assessed.

**Results:**

PHG, defined as random blood glucose ≥10.0 mmol/L (180 mg/dl) on two occasions within 24 h, occurred in 65.28% of cardiac surgery patients. Multivariable logistic regression analysis identified that age [odds ratio (OR) 1.054, 95% confidence interval (CI) 1.040–1.069; *p* < 0.001], female sex (OR 1.380, 95% CI 1.023–1.864; *p* = 0.035), diabetes (OR 13.101, 95% CI 4.057–42.310; *p* < 0.001), pulmonary infection (OR 1.918, 95% CI 1.129–3.258; *p* = 0.016), aortic cross-clamp time (OR 1.007, 95% CI 1.003–1.010; *p* < 0.001), and intraoperative highest glucose (OR 1.515, 95% CI 1.370–1.675; *p* < 0.001) emerged as independent risk factors for PHG. Moreover, PHG had higher rates of acute kidney injury (12.61% vs. 4.00%; *p* < 0.001), delirium (9.57% vs. 3.43%; *p* < 0.001), pulmonary infection (12.01% vs. 5.14%; *p* < 0.001), longer duration of mechanical ventilation (19 vs. 14 h; *p* < 0.001), length of ICU stay (74 vs. 58 h; *p* < 0.001), length of hospitalization (13 vs. 11 days; *p* < 0.001), and higher rate of self-discharge or death (3.95% vs. 0.57%; *p* = 0.002) compared with patients with non-PHG.

**Conclusions:**

PHG occurs frequently in patients after cardiac surgery. Age, female, diabetes, pulmonary infection, aortic cross-clamp time, and intraoperative highest glucose were independent risk factors for PHG. PHG is associated with worse clinical outcomes, including a higher rate of acute kidney injury, delirium, and pulmonary infection, greater duration of mechanical ventilation, length of ICU stay, length of hospitalization, and higher rate of automatic discharge or death.

## Introduction

Cardiac surgery with cardiopulmonary bypass (CPB) is a primary treatment for heart valve disease or coronary artery disease. Globally, approximately 2 million cardiac surgeries using CPB are performed annually (1). In China in 2022, 263,292 cardiac surgeries were conducted, with 159,949 involving CPB (2). During CPB, patients are susceptible to postoperative hyperglycemia (PHG) because of general inflammatory reactions, surgical stressors, hypothermia, catecholamine release, increased catabolism, and the use of corticosteroids or positive inotropic drugs, regardless of preoperative diabetes status (3–5). Hyperglycemia can contribute to endothelial dysfunction, glucose metabolism dysfunction, oxidative stress, inflammation, and mitochondrial dysfunction (6–8).

PHG is defined as blood glucose levels >140 mg/dl (7.8 mmol/L). Its incidence in cardiac surgery is in the range of 60%–80%, with approximately 60% of PHG patients being diagnosed with diabetes mellitus within 1 year of follow up (9). Previous studies have examined the impact of preoperative or intraoperative hyperglycemia on adverse outcomes, such as an increased risk of postoperative infections (10), cognitive dysfunction (11), acute kidney injury (AKI) (12, 13), end-organ dysfunction (14), and death (15).

Several studies have examined the risk factors and outcomes associated with PHG (16–18). Independent risk factors for PHG include higher body mass index (BMI), advanced age, aortic cross-clamp duration, blood transfusion, diabetes, high Euro-SCORE II, and preoperative leukocytosis. Hyperglycemia is also linked to worse clinical outcomes, including a higher incidence of AKI, arrhythmias, intensive care unit (ICU)-acquired weakness, prolonged mechanical ventilation, extended ICU stays, and increased rates of multiorgan failure. In addition, Gillinov et al. (19) reported that non-diabetic cardiac surgery patients with PHG incurred significantly higher hospital costs than those without PHG ($38,642 vs. $28,987). Therefore, postoperative hyperglycemia has become a hot topic in cardiac surgery. Our hospital is the largest cardiac surgery center in southwest China. The aim of the present study was to evaluate postoperative blood glucose levels, risk factors for hyperglycemia, and their association with clinical outcomes in patients undergoing cardiac surgery with CPB.

## Methods

### Study design

This retrospective, observational clinical study included consecutive patients who underwent cardiac surgery with cardiopulmonary bypass at West China Hospital of Sichuan University between January 2023 and March 2024. The study was conducted in accordance with the “Declaration of Helsinki” and was approved by the ethics committee of West China Hospital of Sichuan University (reference number: 2024 Annual Audit 848). Due to the observational and retrospective nature of this study, the requirement for written informed consent was waived.

### Study population

The study included 1,008 consecutive patients who were admitted to the intensive care unit after cardiac surgery during this period. The exclusion criteria were as follows: pregnancy, death during surgery, age <18 years, missing data exceeding 20%, and corticosteroid use. Patients were categorized according to their blood glucose levels: those with blood glucose ≥10.0 mmol/L (*n* = 658) and those with blood glucose <10.0 mmol/L (*n* = 350) for group comparison.

### Data collection

Patients’ clinical data were collected from the electronic medical records system of West China Hospital. Preoperative variables included demographic factors (age, sex, BMI, smoking and drinking history, level of education, marital status), clinical conditions [hypertension, diabetes mellitus, coronary heart disease, stroke, chronic kidney disease, chronic lung disease, pulmonary infection, and history of cardiovascular surgery, New York Heart Association (NYHA) ≥ 3], and laboratory tests [hemoglobin, white blood cell count, neutrophil count, lymphocyte count, red blood cell count, platelet count, hemoglobin, neutrophil percentages, albumin, blood glucose, creatinine, glomerular filtration rate, triglyceride, cholesterol, high-density lipoprotein (HDL), low-density lipoprotein (LDL), left ventricle (LV), and left ventricular ejection fraction (LVEF)]. Operative variables included operation time, CPB time, aortic cross-clamp time, intraoperative highest glucose and lactate levels, and urgency and type of surgery. Postoperative variables included postoperative hyperglycemia, acute kidney injury, delirium, pulmonary infection, duration of mechanical ventilation, ICU and hospital length of stay, self-discharge or death, and 30-day readmission.

### Definition

Postoperative hyperglycemia is defined as a random blood glucose level ≥10 mmol/L on two occasions within 24 h (20, 21). Blood glucose monitoring was performed immediately postoperatively and then every 3–4 h daily up to 10 days or until discharge from the ICU. Blood glucose levels were measured using arterial blood gas analysis and capillary blood glucose monitoring. At our center, when postoperative random blood glucose reaches ≥11.1 mmol/L on two or more occasions, the electronic medical record system issues an alert, prompting nurses to administer intravenous insulin and adjust the dose accordingly. Insulin infusion was discontinued when blood glucose levels dropped below 11.1 mmol/L. AKI was defined according to the Kidney Disease Improving Global Outcomes (KDIGO) (22): (1) an increase in serum creatinine (SCr) by 0.3 mg/dl within 48 h or (2) an increase in SCr by 1.5 times the baseline value within 7 days, or initiation of renal replacement therapy. Serum creatinine levels were measured preoperatively and monitored for 10 days postoperatively. Delirium was assessed daily using the confusion assessment method for the ICU (CAM-ICU) (23). A patient was considered to have developed delirium if they tested positive on the CAM-ICU assessment, which ICU was conducted by ICU nurses six times per day until ICU discharge. Pulmonary infection was recorded in the electronic medical record by the doctor, with diagnoses based on imaging (chest X-ray and CT scans) and examination indicators (e.g., white blood cells).

### Statistical analysis

Statistical analyses were conducted using SPSS version 22.0 (IBM Corp., Armonk, NY, USA). Continuous variables with a normal distribution are presented as mean ± SD, while those with a non-normal distribution are expressed as median (25th–75th percentiles). The Shapiro–Wilk test was used to assess the normal distribution of values. Categorical variables are reported as absolute values and relative frequencies. Univariate logistic regression analysis was performed on all variables to evaluate risk factors for hyperglycemia.

A multivariate logistic regression analysis model (enter method) was also performed to identify independent predictors of hyperglycemia for those variables with statistical significance in the univariate analysis (*p* < 0.05) and to compare clinical outcomes between the PHG and non-PHG groups. A *p*-value of <0.05 was considered statistically significant.

## Results

There were 1,013 patients who underwent cardiac surgery with cardiopulmonary bypass at West China Hospital of Sichuan University between January 2023 and March 2024. Among them, five patients were excluded: one patient was aged <18 years, two patients died within 24 h after surgery, and two patients had missing data. Finally, 1,008 patients were included in this study.

Preoperative and operative variables of these patients are shown in Tables 1 and 2. Of the 1,008 patients, 658 had PHG according to the definition above. The mean age of patients in the non-PHG and PHG groups was 53 and 59 years, respectively. In the non-PHG group, 55.71% of patients were male, compared to 49.24% in the PHG group. The prevalence of diabetes was significantly higher in the PHG group than in the non-PHG group (16.26% vs. 0.86%; *p* < 0.001).

**Table 1:** Preoperative and operative variables of patients in different groups.

Variables	Total (*n* = 1,008)	Non-PHG group (*n* = 350)	PHG group (*n* = 658)	*p*-value
Age (years)	57 (50–64)	53 (46–59)	59 (52–66)	<0.001^a^
Males, *n* (%)	519 (51.49)	195 (55.71)	324 (49.24)	0.050
BMI (kg/m^2^)	23.43 (21.12–25.60)	22.90 (20.72–25.24)	23.52 (21.45–25.81)	0.002^a^
Smoking history, *n* (%)	245 (24.31)	95 (27.14)	150 (22.80)	0.126
Drinking history, *n* (%)	202 (20.04)	78 (22.29)	124 (18.84)	0.194
Educational levels				
Primary and lower, *n* (%)	298 (29.56)	79 (22.57)	219 (33.28)	<0.001^a^
Middle school, *n* (%)	497 (49.31)	177 (50.57)	320 (48.63)	
University and higher, *n* (%)	213 (21.13)	94 (26.86)	119 (18.09)	
Married, *n* (%)	906 (89.88)	310 (88.57)	596 (86.47)	0.013^a^
Hypertension, *n* (%)	251 (24.90)	68 (19.43)	183 (27.81)	0.003^a^
Diabetes, *n* (%)	110 (10.91)	3 (0.86)	107 (16.26)	<0.001^a^
CAD, *n* (%)	187 (18.55)	47 (13.43)	140 (21.28)	0.002^a^
Stroke, *n* (%)	58 (5.75)	12 (3.71)	46 (6.99)	0.021^a^
CKD, *n* (%)	46 (4.56)	13 (3.47)	33 (5.02)	0.346
Chronic lung disease, *n* (%)	52 (5.16)	12 (3.43)	40 (6.08)	0.070
Pulmonary infection, *n* (%)	113 (11.21)	24 (6.86)	89 (13.53)	0.001^a^
Anemia, *n* (%)	25 (2.48)	9 (2.57)	16 (2.43)	0.892
History of cardiovascular surgery, *n* (%)	115 (11.41)	31 (8.86)	84 (12.77)	0.063
NYHA ≥3, *n* (%)	446 (44.25)	140 (40.00)	306 (46.50)	0.048^a^
Hemoglobin (g/L)	135 (121–145)	136 (122–148)	134 (121–144)	0.072
White blood cells (×10^9^/L)	5.95 (4.87–7.17)	5.81 (4.72–6.94)	6.06 (5.00–7.23)	0.024^a^
Neutrophil percentages (%)	59.5 (52.8–66.3)	58.2 (52.5–64.5)	59.8 (53.1–67.6)	0.017^a^
Albumin (g/L)	42.8 (40.0–45.5)	43.3 (40.5–45.9)	42.7 (39.8–45.3)	0.052
Blood glucose (mmol/L)	5.05 (4.63–5.81)	4.89 (4.52–5.36)	5.17 (4.67–6.11)	<0.001^a^
Creatinine (g/L)	78 (66–90)	78 (66–89)	77 (66–92)	0.754
Glomerular filtration rate (ml/min/1.73 m^2^)	86.55 (71.61–97.74)	90.44 (77.72–101.83)	83.67 (69.36–95.75)	<0.001^a^
Triglyceride (mmol/L)	1.23 (0.92–1.67)	1.17 (0.89–1.63)	1.26 (0.94–1.71)	0.025^a^
Cholesterol (mmol/L)	4.20 (3.48–4.92)	4.35 (3.68–4.88)	4.13 (3.39–4.94)	0.082
HDL (mmol/L)	1.21 (0.97–1.47)	1.24 (1.00–1.50)	1.19 (0.96–1.44)	0.151
LDL (mmol/L)	2.54 (1.95–3.10)	2.63 (2.17–3.07)	2.45 (1.87–3.10)	0.008^a^
LV	51 (46–59)	51(46–59)	51(46–59)	0.688
LVEF (%)	63(57–69)	63(59–69)	63(57–69)	0.181

CAD, coronary artery disease; CKD, chronic kidney disease; NYHA, New York Heart Association; HDL, high-density lipoprotein; LDL, low-density lipoprotein; LV, left ventricle; LVEF, left ventricular ejection fraction.

Median (25th–75th percentiles) for variables without normal distribution.

^a^ The statistical significance was set at a *p*-value <0.05.

**Table 2:** Operative variables of patients in different groups.

Variables	Total (*n* = 1,008)	Non-PHG group (*n* = 350)	PHG group (*n* = 685)	*p*-value
Duration of surgery (min)	255 (214–302)	248 (203–290)	260 (220–304)	<0.001
CPB time (min)	126 (95–160)	113 (89–147)	131 (100–163)	<0.001
Duration of aortic cross-clamp, min	91 (64–119)	82 (58–113)	96 (68–122)	<0.001
Intraoperative highest glucose (mmol/L)	7.2 (6.2–8.5)	6.5 (5.7–7.4)	7.6 (6.4–9.2)	<0.001
Intraoperative highest lactate (mmol/L)	2.9 (2.1–3.9)	2.5 (1.9–3.4)	3.1 (2.3–4.2)	<0.001
Urgent surgery, *n* (%)	74 (7.34)	24 (6.86)	50 (7.60)	0.667
Type of surgery				
Valve replacement, *n* (%)	710 (70.44)	244 (69.71)	466 (70.82)	0.002*
Valvuloplasty, *n* (%)	116 (11.51)	44 (12.57)	72 (10.94)	
CABG, *n* (%)	79 (7.84)	17 (4.86)	62 (9.42)	
Cardiac tumor resection, *n* (%)	48 (4.76)	28 (8.00）	20 (3.04)	
Valve surgery + CABG, *n* (%)	21 (2.08)	5 (1.43)	16 (2.43)	
Other surgeries, *n* (%)	34(3.37)	12(3.43)	22(3.34)	

CPB, cardiopulmonary bypass; CABG, coronary artery bypass grafting.

We observed that the mean BMI of patients in the PHG group was significantly higher than that of patients in the non-PHG group (23.52 vs. 22.90; *p* = 0.002). The proportion of patients with a primary or lower education level was significantly higher in the PHG group (33.28% vs. 22.57%; *p* < 0.001). There was also a significant difference in preoperative blood glucose levels between the two groups.

**Figure 1:**
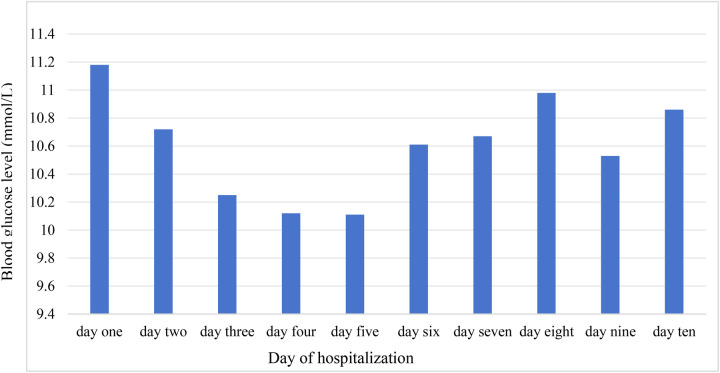
The trend of highest mean blood glucose during the ICU period.

Regarding operative variables, operation time, CPB time, aortic cross-clamp time, and intraoperative highest glucose and lactate levels showed significant differences between groups. During ICU hospitalization, 65.28% of patients had glucose levels ≥10.0 mmol/L. In addition, the mean daily highest blood glucose levels during the ICU period are illustrated in Figure 1.

In multivariable analysis (Table 3), it was determined that age [odds ratio (OR) 1.054, 95% confidence interval (CI) 1.040–1.069; *p* < 0.001], female sex (OR 1.380, 95% CI 1.023–1.864; *p* = 0.035), diabetes (OR 13.101, 95% CI 4.057–42.310; *p* < 0.001), pulmonary infection (OR 1.918, 95% CI 1.129–3.258; *p* = 0.016), aortic cross-clamp time (OR 1.007, 95% CI 1.003–1.010; *p* < 0.001), and intraoperative highest glucose level (OR 1.515, 95% CI 1.370–1.675; *p* < 0.001) were independent risk factors for PHG. We also analyzed the impact of PHG on clinical outcomes in cardiac surgery patients. The results suggest that patients in the PHG group experienced significantly worse postoperative outcomes compared to those in the non-PHG group: AKI = 12.61% vs. 4.00% (*p* < 0.001), delirium = 9.57% vs. 3.43% (*p* < 0.001), pulmonary infection = 12.01% vs. 5.14% (*p* < 0.001), and self-discharge or death = 3.95% vs. 0.57% (*p* = 0.002). Postoperative ventilation time (19 vs. 14 h; *p* < 0.001), length of ICU stay (74 vs. 58 h; *p* < 0.001), and length of hospitalization (13 vs. 11 days; *p* < 0.001) for patients in the PHG group were significantly longer than those of patients in the non-PHG group (Table 4).

**Table 3:** Multivariable analysis of PHG.

Variables	OR	95% CI	*p-*value
Age	1.054	1.040–1.069	<0.001
Female sex	1.380	1.023–1.864	0.035
Diabetes	13.101	4.057–42.310	<0.001
Pulmonary infection	1.918	1.129–3.258	0.016
Duration of aortic cross-clamp time	1.007	1.003–1.010	<0.001
Intraoperative highest glucose	1.515	1.370–1.675	<0.001

**Table 4:** Postoperative complications in different groups.

Complications	Total	Non-PHG group	PHG group	*p*-value
AKI, *n* (%)	97 (9.62)	14 (4.00)	83 (12.61)	<0.001
Delirium, *n* (%)	75 (7.44)	12 (3.43)	63 (9.57)	<0.001
Pulmonary infection, *n* (%)	97 (9.62)	18 (5.14)	79 (12.01)	<0.001
Length of mechanical ventilation (h)	17 (12–22)	14 (11–18)	19 (13–27)	<0.001
Length of ICU stay (h)	68 (45–97)	58 (42–87)	74 (47–113)	<0.001
Length of hospitalization (days)	12 (10–17)	11 (9–15)	13 (10–18)	<0.001
Debridement of chest wounds, *n* (%)	6 (0.60)	1 (0.29)	5 (0.76)	0.671
Unplanned readmission to ICU, *n* (%)	15 (1.49)	4 (1.14)	11 (1.67)	0.509
Self-discharge or death, *n* (%)	28 (2.78)	2 (0.57)	26 (3.95)	0.002
30-day readmission, *n* (%)	17 (1.71)	4 (1.14)	13 (1.98)	0.317

Median (25th–75th percentiles) for variables without normal distribution.

## Discussion

CPB is a standard procedure in cardiac surgery; however, it may trigger several biochemical changes in the microcirculation, leading to a systemic inflammatory response (24). This results from factors such as blood contact with the CPB device's surface, surgical trauma, endotoxemia, blood loss, and ischemic reperfusion injury (25). Systemic inflammation has recently been associated with hyperglycemia and insulin resistance in the adult diabetic population. A recent study reported that inflammation induced by CPB may contribute to insulin resistance, leading to postoperative hyperglycemia (26). In addition, other factors, such as planned hypothermia during surgery, surgical stress, the use of glucocorticoids, and positive inotropes, may disrupt glucose metabolism (27). Therefore, hyperglycemia occurs frequently in patients undergoing cardiac surgery, regardless of their history of diabetes mellitus. It has also been associated with an increased risk of postoperative complications, morbidity, and mortality (18).

This study found that the incidence of hyperglycemia (≥10.0 mmol/L) in cardiac surgery patients was 65.28%. Independent risk factors for PHG included age, female sex, diabetes, pulmonary infection, aortic cross-clamp time, and intraoperative highest glucose levels. Kourek et al. (17) reported that PHG (≥10 mmol/L) occurred in 30% of cardiac surgery patients, with diabetes, high Euro-SCORE II, and preoperative leukocytosis as independent risk factors. Moorthy et al. (16) identified higher BMI, age, aortic cross-clamp time, and blood transfusion as independent risk factors of PHG after cardiac surgery in non-diabetic patients. Chen et al. (18) reported that 42.36% of adult patients undergoing type A aortic dissection developed PHG, with neutrophil count, platelet count, lactic acid levels, weight, and lymphocyte count as predictors. Compared with previous studies (16–18), the incidence of hyperglycemia in our center was significantly higher. A possible explanation is that patients undergoing cardiac surgery at our hospital are started on an intravenous insulin infusion when their blood glucose reaches ≥11.1 mmol/L. Consistent with previous studies, age (16, 28) and female sex (28) were identified as risk factors for PHG, and Kourek et al. (17) demonstrated a sex-related association with PHG levels.

Our study’s novel and most significant finding is that preoperative pulmonary infection and maximum intraoperative glucose are independent risk factors for PHG in patients undergoing cardiac surgery with CPB. The mechanism linking PHG to preoperative pulmonary infection remains unclear. The physiological stress induced by pulmonary infection can lead to metabolic disorders, including altered hepatic glucose metabolism, increased peripheral insulin resistance, and hyperglycemia (29). In patients with pneumonia, elevated C-reactive protein (CRP) have been positively associated with insulin resistance (30). Salonen et al. (31) demonstrated that advanced age, high gHbA1c, elevated CRP, and high blood leukocyte levels are risk factors for hyperglycemia in patients with pneumonia, with inflammation markedly enhancing insulin resistance. Therefore, reducing preoperative pulmonary infections in cardiac surgery patients could reduce the incidence of PHG.

Our study also found that intraoperative blood glucose levels were significantly higher in the PHG group than in the non-PHG group and that the highest intraoperative blood glucose was a risk factor for PHG. In their study, Nair et al. (32) reported that intraoperative hyperglycemia increases the odds for PHG, and Yamamoto et al. (33) found that intraoperative hyperglycemia, longer CPB time, younger age, and chromosomal abnormalities were risk factors for severe PHG (blood glucose ≥250 mg/dl) in pediatric cardiac surgery patients. Intraoperative hyperglycemia increases the risk of acute kidney injury (34), delirium (35), infection (10), and 30-day mortality (36) in patients undergoing cardiac surgery. Currently, there is no definitive consensus on the optimal perioperative blood glucose range for cardiac surgery patients. Current guidelines recommend maintaining blood glucose below 180 mg/dl (10 mmol/L) during CPB (37). In addition, aortic cross-clamp time was associated with PHG, consistent with the research by Moorthy et al. (16). Longer aortic cross-clamp times may increase inflammatory mediators (e.g., IL-6, IL-8, and IL-10), which can predispose patients to hyperglycemia (38).

In this study, patients who experienced PHG had higher rates of kidney injury, delirium, and pulmonary infection, as well as longer durations of mechanical ventilation, ICU stay, hospitalization, and a higher rate of self-discharge or death. Although many studies have examined perioperative blood glucose management in cardiac surgery patients, fewer have focused on PHG. Kourek et al. (17), Moorthy et al. (16), and Chen et al. (18) identified PHG as a strong predictor of AKI, multiorgan failure, hepatic dysfunction, cardiac arrhythmias, and mortality, regardless of diabetes status. Although guidelines recommend keeping postoperative blood glucose below 10 mmol/L (20), a study (39) found that among insulin-treated diabetic patients, maintaining glucose levels in the range of 180–240 mg/dl was associated with cost reductions of $6,225 per patient, a 1.6-day reduction in length of hospital stay, a 4.1% reduction in infections, and a 12.5% reduction in respiratory complications. Therefore, optimal postoperative glycemic management should be further explored in post-cardiac surgery patients.

### Limitations

There are several limitations to this study. First, this is a single-center retrospective study. Second, some studies defined PHG as blood glucose ≥10 mmol/L (16–18), while others define it as blood glucose ≥11.1 mmol/L (40, 41). Even though we used relaxed blood glucose control criteria, our center still followed guideline recommendations (20, 21) and defined hyperglycemia as ≥10.0 mmol/L. Third, patients with diabetes mellitus were not excluded and postoperative insulin use not considered. Finally, unfortunately, variables such as Hba1C and EUROSCORE were not included in our study due to missing values.

## Conclusion

Age, female sex, diabetes, pulmonary infection, aortic cross-clamp time, and intraoperative highest glucose are independent risk factors for PHG. PHG was also significantly associated with the occurrence of AKI, delirium, pulmonary infection, automatic discharge or death, increased ventilation time, and longer ICU and hospital stays. Our study highlights that cardiac surgeons, anesthesiologists, intensive care physicians, and nurses should pay attention to perioperative hyperglycemia and develop prevention and management strategies.

## Data Availability

The original contributions presented in the study are included in the article/Supplementary Material, further inquiries can be directed to the corresponding author.
